# A Systematic Bioinformatics Workflow With Meta-Analytics Identified Potential Pathogenic Factors of Alzheimer’s Disease

**DOI:** 10.3389/fnins.2020.00209

**Published:** 2020-03-13

**Authors:** Sze Chung Yuen, Hongmei Zhu, Siu-wai Leung

**Affiliations:** ^1^State Key Laboratory of Quality Research in Chinese Medicine, Institute of Chinese Medical Sciences, University of Macau, Macao, China; ^2^School of Informatics, College of Science and Engineering, University of Edinburgh, Edinburgh, United Kingdom

**Keywords:** Alzheimer’s disease, meta-analysis, microarray analysis, RNA sequence analysis, bioinformatics

## Abstract

Potential pathogenic factors, other than well-known *APP*, *APOE4*, and *PSEN*, can be further identified from transcriptomics studies of differentially expressed genes (DEGs) that are specific for Alzheimer’s disease (AD), but findings are often inconsistent or even contradictory. Evidence corroboration by combining meta-analysis and bioinformatics methods may help to resolve existing inconsistencies and contradictions. This study aimed to demonstrate a systematic workflow for evidence synthesis of transcriptomic studies using both meta-analysis and bioinformatics methods to identify potential pathogenic factors. Transcriptomic data were assessed from GEO and ArrayExpress after systematic searches. The DEGs and their dysregulation states from both DNA microarray and RNA sequencing datasets were analyzed and corroborated by meta-analysis. Statistically significant DEGs were used for enrichment analysis based on KEGG and protein–protein interaction network (PPIN) analysis based on STRING. AD-specific modules were further determined by the DIAMOnD algorithm, which identifies significant connectivity patterns between specific disease-associated proteins and non-specific proteins. Within AD-specific modules, the nodes of highest degrees (>95th percentile) were considered as potential pathogenic factors. After systematic searches of 225 datasets, extensive meta-analyses among 25 datasets (21 DNA microarray datasets and 4 RNA sequencing datasets) identified 9,298 DEGs. The dysregulated genes and pathways in AD were associated with impaired amyloid-β (Aβ) clearance. From the AD-specific module, Fyn, and EGFR were the most statistically significant and biologically relevant. This meta-analytical study suggested that the reduced Aβ clearance in AD pathogenesis was associated with the genes encoding Fyn and EGFR, which were key receptors in Aβ downstream signaling.

## Introduction

Alzheimer’s disease is a neurodegenerative disease that is the major cause of dementia worldwide (Alzheimer’s Association, 2009). The AD brain is characterized by the distribution of amyloid plaques and neurofibrillary tangles, which are composed of Aβ and hyperphosphorylated tau proteins, respectively (Wang and Mandelkow, 2016; Wang J. et al., 2017). The generation of Aβ from amyloid precursor protein (APP) is a central theme in the field of AD. APP is a single transmembrane protein with a large extracellular domain that is generated in large quantities and efficiently metabolized. APP can be processed via non-amyloidogenic or amyloidogenic generation of Aβ (Chow et al., 2010). Aβ mainly exists in two forms, Aβ_40_ and a more hydrophobic Aβ_42_, which consist of 40 and 42 amino acids, respectively (Marina et al., 2003). The Aβ monomer is intrinsically disordered, and present as a dynamic conformational structure (Chen et al., 2017), which allows the monomer binds to numerous substrate with a high binding affinity. The monomers of Aβ aggregate into oligomers, fibrils, and plaques, when a critical concentration of amyloid is reached due to dysfunctional homeostasis (Knowles et al., 2014). The soluble Aβ oligomer is recognized as the major neurotoxic species, and exists in an equilibrium with fibrils (Yang et al., 2017). And the toxicity of Aβ oligomers is inversely correlated with their oligomer size (Sengupta et al., 2016). Aβ is directly neurotoxic and induces a series of cellular responses; for example, it can induce excitotoxic signaling by increasing glutamate release (Brito-Moreira et al., 2011), and alter Ca^2+^ homeostasis and synaptic functions in neurons, astrocytes, and microglia (Parihar and Brewer, 2010). Aβ located on the mitochondria generates ROS, thus increasing oxidative stress and disrupting oxidative phosphorylation (Caspersen et al., 2005). Aβ also activates protein kinase to phosphorylate tau protein, resulting in the formation of neurofibrillary tangles (Hernández and Avila, 2008). The toxicity of both Aβ and hyperphosphorylated tau proteins are dependent on one another; an absence of tau protein reduces Aβ-induced memory impairment, while increased Aβ levels promote tau pathology (Oddo et al., 2006; Roberson et al., 2007).

The amyloid cascade hypothesis was proposed in 1992, and depicts Aβ as the causative factor in AD development (Hardy and Higgins, 1992). This hypothesis has genetic-based support, because mutations in genes related to Aβ formation (*APP* and *PSEN*) and clearance (*APOE4*) induce early-onset AD (Rovelet-Lecrux et al., 2006; Kline, 2012; St George-Hyslop and Fraser, 2012). However, this hypothesis is challenged by two facts, although there are also counterarguments that have been proposed from other scientific findings. First, amyloid imaging reveals that Aβ deposition also occurs in healthy aging subjects, suggesting that Aβ deposition could be a normal phenomenon of aging (Edison et al., 2007). However, Esparza et al. (2013) suggested that Aβ oligomer levels, which are the neurotoxic form of Aβ deposition, are higher in AD brains compared with healthy subjects. Second, the degree of dementia does not correlate well with amyloid plaque formation in AD (Nelson et al., 2012). In fact, Aβ oligomers may exert their neurotoxic effects much earlier, before the formation of amyloid plaques (Broersen et al., 2010). Also, the drug development for the treatment of AD has been guided by the amyloid cascade hypothesis for the pass two decade, although most anti-Aβ drugs fail in clinical trials and no novel drugs have been brought to market since 2003 (Cummings et al., 2014). For example, Solanezumab (currently in a phase 3 trial) recognizes Aβ at the 13 to 28 amino acid position and lowers amyloid levels, but does not show beneficial effects in subjects with mild AD (Honig et al., 2018). Verubecestat (currently in a phase 3 trial), a β-secretase inhibitor, reduces Aβ levels in a dose-dependent manner (Kennedy et al., 2016). However, a recent clinical trial (Egan et al., 2019) indicated that verubecestat does not have clinical effects in AD subjects. The failure of development of anti-Aβ drugs does not discredit amyloid as the central theme for AD treatment, because the sporadic AD is caused by the changes of genes which potentiate the neurotoxicity of Aβ in the brain over years, rather than solely Aβ overproduction. Indeed, the cerebral Aβ_42_ kinetics were modified by Aβ accumulation (Potter et al., 2013), resulting in the disease preventative treatment required at least 95% lowering Aβ_42_ production if the treatment was started after Aβ accumulation (Roberts et al., 2017). This suggests that the development of anti-Aβ drugs should not be solely dependent on inhibition of Aβ_42_ production. However, the incomplete knowledge of AD pathogenic factors may hindered the investigation of AD (Scott et al., 2014), not just Aβ production, but also the Aβ-relevant receptors and their corresponding downstream signaling cascades.

Alzheimer’s disease is a complex disorder, expressed as a malfunction of defects in multiple genes. The discovery of AD pathogenic factors can be revealed by transcriptome profiling approach [i.e., DNA microarray and RNA-Seq (Sutherland et al., 2011)] to identify DEGs in AD subjects compared with healthy subjects. DEGs are genes that are potentially associated with disease pathology. Table 1 shows the publications using transcriptomic data and bioinformatics tools for identifying AD pathogenic factors. Puthiyedth et al. (2016) identified common DEGs from microarray datasets across six different brain regions. All datasets were integrated using the Colored (α, β)-κ Feature set approach (Puthiyedth et al., 2015), and a few common DEGs were identified across the brain regions, revealing an AD-specific signature. Kawalia et al. (2017) used the BC3Net10 algorithm to infer AD gene regulatory networks. This network was generated by the integration of literature-based knowledge and data-driven analysis. Inferences did not solely depend on the network topology, but also integrated information about DEGs and AD-related genes, after systematic searching from databases. However, the DEGs obtained from different transcriptomic studies of AD are sometimes conflicting because of many factors, such as the use of different experimental designs or statistical methods between studies. The integration of these studies based on meta-analysis therefore resolves inconclusive results and obtains a generalization (Haidich, 2010). The results of meta-analysis are presented as logORs based on the number of dysregulation events in both disease and control samples. The effect sizes are integrated to assess the overall effect size based on either fixed- or random-effect models. Meta-analysis has been applied in genomic analyses to assess the effectiveness of a particular gene in AD. Moradifard et al. (2018) conducted a meta-analysis using the R package *RobustRankAggreg* to identify statistically significant DEGs in AD across six microarray datasets. The DEGs were further corroborated with three RNA-Seq datasets and input for biological enrichment analysis to reveal the miRNAs that regulate the DEGs. Furthermore, Wang Q. et al. (2017) conducted a meta-analysis using the R package *RankProd* to explore the molecular mechanisms of AD across seven microarray datasets. They found 37 DEGs commonly shared by six brain regions, and most of these DEGs were downregulated. The significantly enriched pathways of DEGs were associated with mitochondrial oxidative phosphorylation and synaptic vesicle function. However, both studies only focused on a single microarray platform (Affymetrix), and the meta-analysis of DEGs did not include the gene expression profiles from RNA-Seq. For the study by Moradifard et al., the DEGs from RNA-Seq were only used to validate the results of microarray datasets; that is, the DEGs common to both the RNA-Seq and microarray datasets, in the same dysregulated direction. It seems that the results of currently available meta-analyses of DEGS for identifying AD pathogenic genes are limited by the use of microarray platforms and the inability to integrate results from both microarray and RNA-Seq.

**TABLE 1:** The published transcriptomic data and bioinformatics tools for identifying AD pathogenic factors.

Title	Authors (publication year)	Database	Bioinformatic tools	Pathogenic factors
Integrated Identification of Key Genes and Pathways in Alzheimer’s Disease via Comprehensive Bioinformatical Analyses (Yan et al., 2019)	Yan et al. (2019)	GEO, KEGG, Reactome, STRING, Wikipathway	Platform: Morpheus, RBPDB, UCSC; Software: Cytoscape, ClueGO, Gluepedia, Graphpad Prism, MCODE	*BDNF*, *CACNA1A*, *CALB*, *CD44*, *CDC42*, *OXT*, *PDYN*, *TAC1*, *TH*, *VEGFA*
Systematic Analysis and Biomarker Study for Alzheimer’s Disease (Li X. et al., 2018)	Li X. et al. (2018)	GEO, International Genomics of Alzheimer’s Project	R package: *affy*, *glmnet*, *limma*, *MASS*, *PRROC*, *ROCR*; Software: Ingenuity Pathway Analysis, MAGMA	*NDUFA1*, *MRPL51*, *RPL36AL*
Network-based approach to identify molecular signatures and therapeutic agents in Alzheimer’s Disease (Rahman et al., 2019)	Rahman et al. (2019)	CMap, GEO, JASPAR, miRTarBase, STRING, TarBase	Platform: DAVID, GEO2R; Software: CytoHubba, Cytoscape, MCODE	*AR*, *CREBBP*, *E2F1*, *FOXC1*, *FOXL1*, *GATA2*, *JUN*, *NFIC*, *PPARG*, *RAC1*, *RPL12*, *RPL15*, *RPS11*, *RPS6*, *SMAD3*, *SRF*, *UBA52*, *UBC*, *USF2*, *YY1*
Alzheimer’s Disease Master Regulators Analysis: Search for Potential Molecular Targets and Drug Repositioning Candidates (Vargas et al., 2018)	Vargas et al. (2018)	CMap, GEO	Algorithm: Algorithm for the Reconstruction of Accurate Cellular Networks, two-tail gene set enrichment analysis; R package: *ggplot2*, *RedeR*, *RTN*	*ATF2*, *CNOT7*, *CSRNP2*, *PARK2*, *SLC30A9*, *TSC22D1*
Condition-specific Gene Co-expression Network Mining Identifies Key Pathways and Regulators in the Brain Tissue of Alzheimer’s Disease Patients (Xiang et al., 2018)	Xiang et al. (2018)	Allen Brain Institute, GEO	Algorithm: Local maximized Quasi-Clique Merger; Platform: REViGO; R package: *Affy*, *Enrichr*, *lmQCM*	*FOS, JUN, MEF2A, MIB2, PCBP1, SMARCA2, SP1, STAT1, TEAD4, ZFHX3, ZNF281*
Analytical Strategy to Prioritize Alzheimer’s Disease Candidate Genes in Gene Regulatory Networks Using Public Expression Data (Kawalia et al., 2017)	Kawalia et al. (2017)	ArrayExpress, CPDB, ENSEMBL, GEO, GWAScatalog, GWASCentral, GWASdb, KEGG, NeuroTransDB, RegulomeDB	Algorithm: BC3Net10; R package: *affy*, *arrayQualityMetrics*, *bc3net*, *limma*; Platform: HaploReg, SCAIView	*AP2A2, ARAP3, ATP2A3, ATP2B4, HLA-C, HLA-F, ITPR2, RAB11FIP4, STX2*
The Bioinformatic Analysis of the Dysregulated Genes and MicroRNAs in Entorhinal Cortex, Hippocampus, and Blood for Alzheimer’s Disease (Pang et al., 2017)	Pang et al. (2017)	CMap, GEO, KEGG	R package: *affy*, *edgeR*, *limma*, *WGCNA*; Platform: DAVID; Software: CytoHubba, Cytoscape, GSEA	*CTSD*, *VCAM1*
A Systematic Integrated Analysis of Brain Expression Profiles Reveals *YAP1* and Other Prioritized Hub Genes as Important Upstream Regulators in Alzheimer’s Disease (Xu et al., 2018)	Xu et al. (2018)	GEO	R package: *in silico Merging*, *limma*, *WGCNA*	*YAP1*
Network Topology Analysis of Post-Mortem Brain Microarrays Identifies More Alzheimer’s Related Genes and MicroRNAs and Points to Novel Routes for Fighting with the Disease (Chandrasekaran and Bonchev, 2016)	Chandrasekaran and Bonchev (2016)	ArrayExpress, GEO, OMIN, ResNet	Algorithm: Robust Multiarray Average approach, empirical Bayes method; Platform: DAVID; Software: Pajek, Pathway Studio	*CD4*, *DCN*, *IL8*
A Systematic Investigation into Aging Related Genes in Brain and Their Relationship with Alzheimer’s Disease (Meng et al., 2016)	Meng et al. (2016)	Biocarta, DisGeNet, GEO, GenAge, GenMapP, MetaBase	Algorithm: Condition-specific target prediction; Platform: DAVID, oPOSSUM; R package: *WGCNA*; Software: Ingenuity Pathway Analysis	*ESR1*, *SOX2*, *SP1*
Identification of Differentially Expressed Genes through Integrated Study of Alzheimer’s Disease Affected Brain Regions (Puthiyedth et al., 2016)	Puthiyedth et al. (2016)	GEO	Algorithm: (α,β)-k Feature Set approach, Minimum Description Length Principle; R package: *GeneMeta*, *RankProd*; Software: Expression Analysis Systematic Explorer	*FGF*, *GPHN*, *INFAR2*, *LARGE*, *PSMB2*, *PSMD14*, *PTMA*, *RAB2A*, *RPL15*, *RWDD2A*, *SEMA4C*, *WNK1*
Meta-Analysis of Transcriptome Data Related to Hippocampus Biopsies and iPSC-Derived Neuronal Cells from Alzheimer’s Disease Patients Reveals an Association with FOXA1 and FOXA2 Gene Regulatory Networks (Wruck et al., 2016)	Wruck et al. (2016)	GEO, KEGG	Platform: oPOSSUM; R package: *affy*, *Gostats*, *lumi*, *oligo*	*FOXA1*, *FOXA2*
A Computational Framework for the Prioritization of Disease-gene Candidates (Browne et al., 2015)	Browne et al. (2015)	BIND, BioGPS, BioGRID, DIP, GEO, HPRD, InACT, MINT, PDB	Algorithm: Average of Pearson correlation coefficients; Platform: hORFeome; R package: *GOSemSim*; Software: Cytoscape, Significance Analysis of Microarrays	*CARD9*, *FHL3*, *KRT38*, *LZTS2*, *MID2*, *MTUS2*, *REL*, *TFCP2*, *TRAF1*
Identification of Unstable Network Modules Reveals Disease Modules Associated with the Progression of Alzheimer’s Disease (Kikuchi et al., 2013)	Kikuchi et al. (2013)	BioGRID, GEO	Algorithm: Infomap algorithm, MAS algorithm	*UCHL5*

*R packages and genes are in italic font.*

**FIGURE 1:**
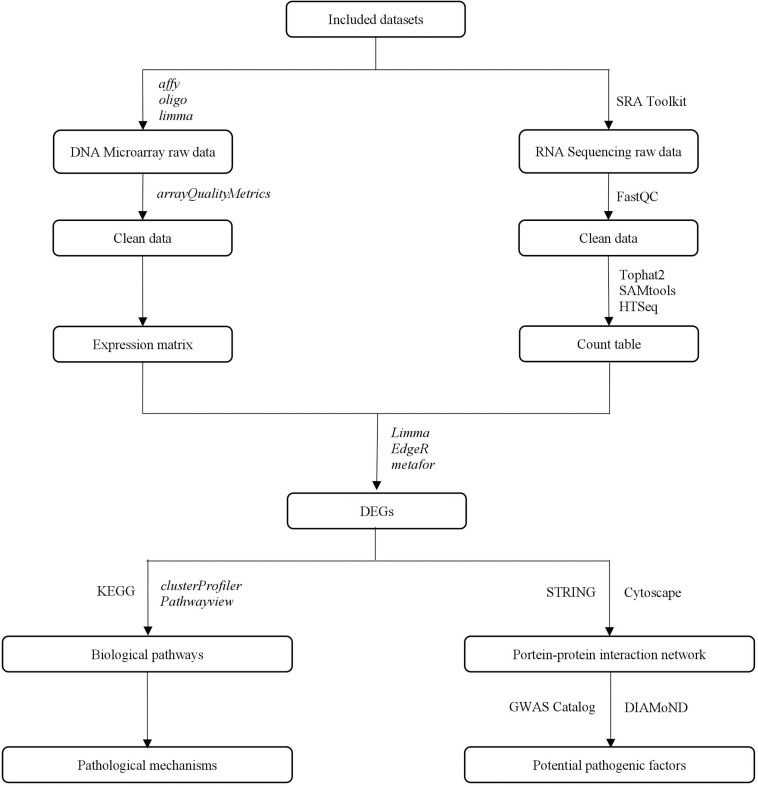
The overall study design.

This study aimed to identify potential pathogenic factors based on the meta-analysis of transcriptomic data from different brain regions in AD. The meta-analysis was conducted according to PRISMA guidelines (Moher et al., 2009), including those for dataset search, dataset selection, and statistical analysis. The raw transcriptomic data were retrieved from databases after a systematic search and were analyzed using a consistent workflow, from quality control and normalization to DEG determination. A statistical meta-analysis was performed when more than one study reported the same DEG. The DEGs that remained statistically significant after *P*-value adjustment were collected to perform a biological enrichment analysis and subgroup analysis, and to construct a PPIN. The network information and AD seed genes, which were obtained from the GWAS Catalog (Welter et al., 2014), were collected for the DIseAse Module Detection (DIAMoND) algorithm (Ghiassian et al., 2015) to examine the significance of the connectivity patterns among network components. This algorithm identified an AD-specific module from the network, and the nodes within the module with a high degree were treated as potential pathogenic factors. The overall study design is illustrated in Figure 1.

## Materials and Methods

### Database Search and Dataset Selection

The datasets were collected from GEO^1^ (Barrett et al., 2012) and ArrayExpress^2^ (Kolesnikov et al., 2015) from their respective inception dates until January 6, 2019. The search strategy is shown in Supplementary File S1.

Raw DNA microarray and RNA-Seq data reporting the transcriptomes of brain regions from AD and healthy subjects were included. The datasets were excluded if they were (1) not case–control studies; (2) focused on other neurodegenerative diseases; (3) demonstrated treatment effects on AD; or (4) used cell-line or artificial AD models. Sample details were collected if they were provided. If the datasets contained several comparisons, DEGs were determined from each comparison independently.

### Data Processing

Each microarray dataset was processed consistently and independently using R packages from Bioconductor. Each dataset was processed through background correction, quantile normalization, and log_2_-transformation of the averaged expression value of duplicate probes, using the *affy* (Gautier et al., 2004), *oligo* (Carvalho and Irizarry, 2010), or *limma* (Ritchie et al., 2015) package. For each dataset, the quality of each array was assessed using the *arrayQualityMetrics* (Kauffmann et al., 2009) package. The array was omitted from the subsequent analysis if it was detected as an outliner in any of the three metrics provided by the package. The gene expression ratios between AD and healthy subjects were determined for each dataset using the *limma* package. Genes with an adjusted *P*-value (by FDR) of less than 0.05 were considered to be statistically significant DEGs. The DEGs were annotated using the gene annotation table provided by GEO and ArrayExpress. The probes with missing gene symbols were removed. When multiple probes were annotated with the same gene symbol on the same array, the probe with the highest expression value was kept.

The raw sequence data were converted to FASTQ format using the SRA Toolkit (version 2.9.2) (Leinonen et al., 2011). Quality control on FASTQ was conducted using FastQC (version 0.11.8) (Andrews, 2010): (1) sequences with less than 80% of bases with at least quality of 25 were filtered; and (2) the bases with quality less than 25 were trimmed. The cleaner FASTQ files were aligned to the *Homo.sapiens* reference genome, GRCh38.94 (downloaded from Ensemble), using TopHat (version 2.1.1) (Kim et al., 2013). The aligned sequences were sorted based on the aligned position to the reference genome using SAMtools (version 1.9) (Li H. et al., 2009). After sorting, the count-based gene expression was obtained using HTSeq (version 0.8.0) (Anders et al., 2015), and the reads with alignment scores less than 10 were omitted. Differential expression was performed using the *edgeR* (Robinson et al., 2009) package for R software. RNA-Seq datasets were processed on a Linux-based HP ProLiant DL580 Gen8 workstation [Inter^®^ Xeon^®^ E7-4820 CPU v2 @ 2.00 GHz; 4 processors with 32 total cores enabled; 128 GB RAM].

### Meta-Analysis, Biological Enrichment Analysis, and Subgroup Analysis

The list of DEGs and their dysregulation states (i.e., upregulated or downregulated) from each study were processed using the *meta* for Chung et al. (2015) package for R software. The meta-analysis was conducted under a random-effects model, and their outcomes were logORs with *P*-values. For each gene in the i-th study, the effect (θ*_i_*) based on the numbers of dysregulation events in both AD and control samples was first calculated, then the overall effect was computed according to formula $\frac{\sum W\,i\,\theta \,i}{\sum W\,i}$, where w_i_ is the weight and is equal to 1/v_i_, where v_i_ is the sample variance. The genes with logORs above or below 0 were considered upregulated or downregulated, respectively. The *P*-values were adjusted by FDR, and DEGs with adjusted *P*-values less than 0.05 were regarded as statistically significant. After meta-analysis, the statistically significant DEGs were used to perform a biological enrichment analysis. The enrichment analysis was conducted by the hypergeometric test using the *clusterProfiler* (Yu et al., 2012) package for R software, based on the KEGG^3^ (Kanehisa et al., 2012). The statistically significant DEGs after meta-analysis were collected as input for the enrichment analysis to identify biological pathways among them. The pathways with FDR-adjusted *P*-values less than 0.05 were considered statistically significant.

The DEGs were split into different subgroups based on which brain regions they were from, to compare expression profiles among brain regions. According to Roth et al. (2006), brain regions can be hierarchically clustered into three major branches based on cytological differences. The first branch includes the cerebellum. The second branch is sub-categorized into three sub-branches; the first sub-branch includes the putamen and nucleus accumbens, the second sub-branch includes the amygdala and hippocampus, and the third sub-branch includes the cerebral neocortex. The third branch includes the thalamus, brain stem, and spinal cord. Biological enrichment analysis was also conducted in each subgroup.

### Protein–Protein Interaction Network and Potential Pathogenic Factors

After meta-analysis, statistically significant DEGs were used to construct a PPIN based on the data from STRING (version 11^4^ ; Szklarczyk et al., 2011). Only interactions with the highest confidence (0.9) were kept. The PPIN was visualized using Cytoscape (Shannon et al., 2003). Disease-associated genes are not topologically interacted into a dense network module because disease can be the consequence of perturbation in many functional units. AD seed genes were retrieved from the GWAS Catalog (Welter et al., 2014)^5^ using the key word “AD” and the following selection criteria: a significance *p*-Value cutoff of no more than 1^∗^10^–8^. Genes with official gene symbols were kept as AD seed genes. The PPIN information (e.g., edge list in the co-expression network) and AD seed genes were put into the DIAMOnD algorithm (Ghiassian et al., 2015) to evaluate the significance of the connections that each node had with the seed genes in the biological network. In each iteration until the stopping condition was satisfied, the node with the lowest connectivity *P*-value was treated as the most significantly connected node for output. The nodes within the module that had a high degree (>95th percentile) were treated as potential pathogenic factors.

### Risk of Bias

All studies were collected according to the publication information of datasets. If there was more than one publication for one dataset, the publication with the earlier publication year was selected. The risk of bias for the included studies was evaluated according to MIAME (Minimum information about a microarray experiment) (Brazma, 2009) and MINSEQE (Minimum information about a high-throughput nucleotide sequencing experiment^6^, proposed by FGED Society in 2012) guidelines by two authors (SY and HZ), independently. Disagreements between the authors were resolved by discussion with the third author (SWL). The MIAME and MINSEQE guidelines are designated to report the quality of DNA microarray and RNA-Seq studies, respectively. And the quality of transcriptomic parts of included studies was assessed, including raw data collection, result presentation, sample annotation, experimental design, array annotation, and data processing pipeline. Items with low risk were counted +1, suggesting high reproducibility; items with unclear risk were counted 0, suggesting ambiguous reproducibility; and items with high risk were counted –1, suggesting low reproducibility.

## Results

### Included Datasets

**FIGURE 2:**
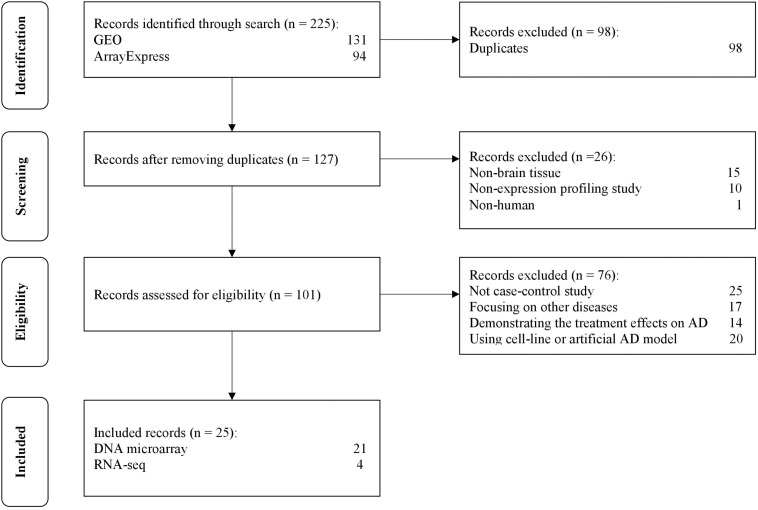
The flow diagram of datasets selection, including identification, screening, eligibility and inclusion stage.

The selection process of the datasets is shown in Figure 2. A total of 225 datasets were initially identified from GEO and ArrayExpress. After removing duplicated datasets and datasets that met the exclusion criteria, 25 datasets (21 for microarray and 4 for RNA-Seq) met the eligibility criteria. The dataset characteristics are shown in Table 2. Among the included datasets, the hippocampus (*n* = 8) was the most extensively studied brain region, followed by the entorhinal cortex and prefrontal cortex (*n* = 3).

**TABLE 2:** The characteristics of included datasets.

Types	Dataset_ID	Brain tissue	Disease stage	Platform	Number of samples	Number of samples omitted
DNA microarray	E-MEXP-2280	Medial temporal lobe	Braak stage VI	Affymetrix Human Genome U133 Plus 2.0 Array	12	0
	GSE110226	Choroid plexus	Braak stage III to VI	Rosetta/Merck Human RSTA Custom Affymetrix 2.0 microarray	13	3
	GSE12685	Frontal cortex	MMSE 21 to 27	Affymetrix Human Genome U133A Array	14	3
	GSE1297	Hippocampus	Braak stage at III to VI	Affymetrix Human Genome U133A Array	31	3
	GSE16759	Parietal lobe cortex	Braak stage at V to VI	Affymetrix Human Genome U133 Plus 2.0 Array	8	1
	GSE26927	Entorhinal cortex	–	Illumina humanRef-8 v2.0 expression beadchip	18	4
	GSE28146	Hippocampus	Braak stage at V to VI	Affymetrix Human Genome U133 Plus 2.0 Array	30	1
	GSE29378	Hippocampus	Braak stage at V to VI	Illumina HumanHT-12 V3.0 expression beadchip	63	2
	GSE32645	Cortex	Braak stage VI	Agilent-014850 Whole Human Genome Microarray 4 × 44K G4112F	6	0
	GSE33000	Prefrontal cortex	–	Rosetta/Merck Human 44k 1.1 microarray	467	30
	GSE36980	Frontal cortex, hippocampus, temporal cortex	Braak stage at V to VI	Affymetrix Human Gene 1.0 ST Array [transcript (gene) version]	79	8
	GSE37263	Neocortex	Braak stage at III to VI	Affymetrix Human Exon 1.0 ST Array [transcript (gene) version]	16	1
	GSE39420	Posterior cingulate	Braak stage at V to VI	Affymetrix Human Gene 1.1 ST Array [transcript (gene) version]	21	0
	GSE44768	Cerebellum	–	Rosetta/Merck Human 44k 1.1 microarray	230	10
	GSE44770	Prefrontal cortex	–	Rosetta/Merck Human 44k 1.1 microarray	230	28
	GSE44771	Visual cortex	–	Rosetta/Merck Human 44k 1.1 microarray	230	22
	GSE48350	Entorhinal cortex, hippocampus, post-central gyrus, superior frontal gyrus	Braak stage at II to VI	Affymetrix Human Genome U133 Plus 2.0 Array	253	16
	GSE5281	Entorhinal cortex, hippocampus, medial temporal gyrus, posterior cingulate, primary visual cortex, superior frontal gyrus	–	Affymetrix Human Genome U133 Plus 2.0 Array	161	11
	GSE61196	Choroid plexus	Braak stage at III and VI	Agilent-014850 Whole Human Genome Microarray 4 × 44K G4112F	21	1
	GSE84422	Amygdala, anterior cingulate, caudate nucleus, dorsolateral prefrontal cortex, frontal pole, hippocampus, inferior frontal gyrus, inferior temporal gyrus, middle temporal gyrus, nucleus accumbens, occipital visual cortex, parahippocampal gyrus, posterior cingulate cortex, precentral gyrus, prefrontal cortex, putamen, temporal polesuperior parietal lobule, superior temporal gyrus	Braak stage at I to VI	Affymetrix Human Genome U133A ArrayAffymetrix Human Genome U133B ArrayAffymetrix Human Genome U133 Plus 2.0 Array	1,146	64
	GSE93885	Olfactory bulb	–	Affymetrix Human Gene 2.0 ST Array [transcript (gene) version]	18	0
RNA-Seq	GSE104704	Lateral temporal lobe	Braak stage at V to VI	Illumina HiSeq 2500 (Homo sapiens)	30	–
	GSE95587	Fusiform gyrus	Braak stage at III to VI	Illumina HiSeq 2500 (Homo sapiens)	117	–
	GSE53697	Dorsolateral prefrontal cortex	Braak stage at II to VI	Illumina HiSeq 2500 (Homo sapiens)	17	–
	GSE67333	Hippocampus	Braak stage at V to VI	Illumina HiSeq 2000 (Homo sapiens)	8	–

### Preprocessing of Datasets and Determination of Differentially Expressed Genes

After the selection process of the datasets, all included datasets contained complete transcriptomic raw data, and were undergone a quality control as a prioritization procedure. In the included microarray datasets, the quality of the normalized dataset was assessed using the R package *arrayQualityMetrics*. Different numbers of arrays were identified as outliers and omitted in the subsequent analysis (Table 2). In the included RNA-Seq datasets, the reads were mapped to the *Homo.sapiens* reference genome (GRCh38.94) after sequence quality assessment. The mapping rates were above 95% for the RNA-Seq datasets. From the mapped reads, the genes were counted to determine DEGs. After preprocessing steps, different numbers of DEGs were determined from each comparison, ranging from 0 to over 10,000 (Table 3). There were 21,064 DEGs in all comparisons that compared AD brain samples with healthy brain samples, and 16,810 DEGs (79.76%) were reported in at least two comparisons (Supplementary File S2). In the meta-analysis of DEGs, 9,298 DEGs were found to be statistically significant (Supplementary File S3); 4,960 genes were downregulated and 4,338 genes were upregulated. The most reported downregulated DEGs were *DPP6* and *FXYD7*, which were reported in 16 comparisons; *RHOQ* was the most reported upregulated DEG among all 15 reported comparisons. A loss of *DPP6* or *FXYD7* is reported to dysregulate neuronal excitation (Hoos et al., 2013; Cacace et al., 2019), while *RHOQ* is reported to enhance Aβ oligomerization (Aguilar et al., 2017). Multiple DEGs involved in Aβ clearance were found statistically significant in meta-analysis (Table 4). These DEGs could be categorized into UPS, autophagy, and UPR, implying the downregulated Aβ clearance. The statistically significant DEGs in UPS and autophagy were mostly downregulated, while those in UPR were mostly upregulated, proposing a dysfunctional homeostasis. The impaired Aβ clearance resulting from dysfunctional homeostasis is suggested to induce Aβ accumulation in sporadic AD, in contrast with the overproduction of Aβ in familial AD (Mawuenyega et al., 2010; Potter et al., 2013).

**TABLE 3:** The number of DEGs determined from each comparison.

Comparison_ID	No of	No of	No of
	control	disease	DEGs
E-MEXP-2280	5	7	0
GSE32645	3	3	0
GSE110226	5	5	475
GSE61196_CvsBraak3	7	7	1,597
GSE61196_CvsBraak6	7	6	1,723
GSE33000	154	283	12,742
GSE12685	6	5	2
GSE1297_CvsIni	8	6	0
GSE1297_CvsMod	9	8	0
GSE1297_CvsSev	9	6	7
GSE93885_CvsIni	4	5	0
GSE93885_CvsMod	4	4	0
GSE93885_CvsSev	4	5	0
GSE29378_CA1	16	15	2
GSE29378_CA3	16	14	1
GSE26927	4	10	0
GSE16759	3	4	0
GSE37263	8	7	0
GSE48350_EC	36	14	7,185
GSE48350_HIP	43	18	3,958
GSE48350_PCG	39	22	1,540
GSE48350_SFG	46	19	6,148
GSE28146_CvsIni	8	7	0
GSE28146_CvsMod	8	8	0
GSE28146_CvsSev	8	6	0
GSE5281_EC	12	9	9,921
GSE5281_HIP	13	10	7,176
GSE5281_MTG	11	16	9,981
GSE5281_PC	12	8	8,978
GSE5281_PVC	12	16	38
GSE5281_SFG	22	9	5,054
GSE44768	98	122	1,447
GSE44770	81	121	1,778
GSE44771	89	119	4,732
GSE84422_Amygdala	14	16	1,253
GSE84422_Nucleus_accumbens	12	16	9
GSE84422_96_Anterior_cingulate	16	20	0
GSE84422_96_Caudate_nucleus	11	18	0
GSE84422_96_Dorsolateral_prefrontal_cortex	15	15	0
GSE84422_96_Frontal_pole	14	21	0
GSE84422_96_Hippocampus	10	18	0
GSE84422_96_Inferior_frontal_gyrus	11	17	0
GSE84422_96_Inferior_temporal_gyrus	13	18	0
GSE84422_96_Middle_temporal_gyrus	14	19	0
GSE84422_96_Occipital_visual_cortex	11	13	0
GSE84422_96_Parahippocampal_gyrus	14	21	0
GSE84422_96_Posterior_cingulate_cortex	12	23	0
GSE84422_96_Precentral_gyrus	5	18	0
GSE84422_96_Prefrontal_cortex	11	18	35
GSE84422_96_Putamen	9	17	0
GSE84422_96_Superior_parietal_lobule	13	12	0
GSE84422_96_Superior_temporal_gyrus	14	20	0
GSE84422_96_Temporal_pole	14	18	0
GSE84422_97_Anterior_cingulate	14	19	0
GSE84422_97_Caudate_nucleus	9	16	0
GSE84422_97_Dorsolateral_prefrontal_cortex	16	14	1
GSE84422_97_Frontal_pole	15	22	0
GSE84422_97_Hippocampus	10	18	0
GSE84422_97_Inferior_frontal_gyrus	11	17	0
GSE84422_97_Inferior_temporal_gyrus	13	18	30
GSE84422_97_Middle_temporal_gyrus	13	20	0
GSE84422_97_Occipital_visual_cortex	12	13	0
GSE84422_97_Parahippocampal_gyrus	14	22	0
GSE84422_97_Posterior_cingulate_cortex	12	22	0
GSE84422_97_Precentral_gyrus	5	17	0
GSE84422_97_Prefrontal_cortex	11	19	0
GSE84422_97_Putamen	9	18	0
GSE84422_97_Superior_parietal_lobule	13	13	0
GSE84422_97_Superior_temporal_gyrus	14	20	1
GSE84422_97_Temporal_pole	14	18	0
GSE36980_FC	17	13	0
GSE36980_HIP	10	5	587
GSE36980_TC	17	9	5
GSE104704_Old	10	12	1,070
GSE95587	33	84	6,611
GSE53697	8	9	1
GSE67333	4	4	10

**TABLE 4:** The dysregulated status of genes involved in ubiquitin-proteasome system, autophagy, and unfolded-protein response for Aβ clearance.

Functional category	Genes	Adjusted *P*-value	Dysregulated state
Ubiquitin	*PRKN*	1.77E-06	Downregulated
-proteasome	*UCHL1*	2.70E-02	Downregulated
system (UPS)	*UCHL3*	1.47E-20	Downregulated
	*PSMC1*	2.09E-24	Downregulated
	*UBE2D2*	2.02E-24	Downregulated
	*STUB1*	1.62E-18	Downregulated
	*NEDD8*	1.88E-31	Downregulated
	*ATXN1*	0.61	Downregulated
	*ATXN3*	0.79	Upregulated
	*ATXN7*	0.97	Upregulated
Autophagy	*BECN1*	1.42E-20	Downregulated
	*MTOR*	1.16E-15	Downregulated
	*TFEB*	2.06E-36	Upregulated
	*ATG5*	5.20E-19	Downregulated
	*ATG7*	1.78E-32	Downregulated
	*TREM2*	5.06E-32	Upregulated
	*MS4A4A*	8.77E-22	Upregulated
	*PLCG2*	6.91E-26	Upregulated
	*ABI3*	1.83E-19	Upregulated
	*ATG12*	0.94	Downregulated
	*PICALM*	0.86	Upregulated
	*SQSTM1*	0.20	Upregulated
Unfolded	*ERN1*	1.05E-13	Upregulated
-protein	*EIF2AK3*	2.00E-10	Upregulated
response (UPR)	*TMEM259*	6.57E-11	Downregulated
	*XBP1*	2.95E-14	Upregulated

However, several genes involved in Aβ clearance were not found statistically significant in meta-analysis, although they were reported as DEGs in separated comparisons. The heat shock proteins work coordinately with UPS for protein clearance, remodeling the misfolded proteins before the degradation by UPS. Both systems are upregulated to restore dysfunctional homeostasis at the beginning (Kästle and Grune, 2012). However, the genes for most heat shock proteins, such as *ATXN1*, *ATXN3*, and *ATXN7*, were not statistically significant (Supplementary File S3), suggesting that the early event of AD might not be truly reflected. *PICALM* and *SQSTM1* which are involved in autophagosome formation (Moreau et al., 2014; Ntsapi and Loos, 2016), were not reported as DEGs in the meta-analysis. Several autophagic marker genes, *MAP1LC3B* and *ATG12*, associated with autophagosomal function (Ma et al., 2010), were also found as a statistically insignificant in the meta-analysis. The expression of *TREM2* was upregulated, which was contradictory to the role of *TREM2* reported in autophagy (Lucin et al., 2013), but consistent with that reported in Aβ plaque-activated microglia (Yuan et al., 2016; Yin et al., 2017). Meanwhile, those genes involved in Aβ plaque-activated microglia, *MS4A4A*, *PLCG2*, and *ABI3*, were also found significantly upregulated in the meta-analysis.

**FIGURE 3:**
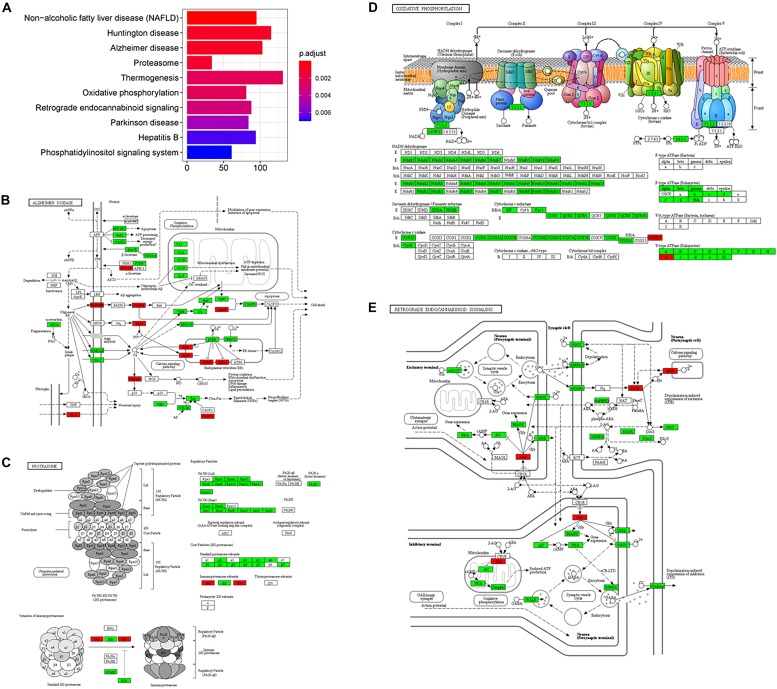
The AD-related biological pathways as impacted in health vs. AD subjects, **(A)** the top 10 statistically significant pathways from enrichment analysis result; **(B)** Alzheimer’s disease; **(C)** Proteasome; **(D)** Oxidative phosphorylation; and **(E)** Retrograde endocannabinoid signaling. For figure 3B-E, the red and green represent upregulation and downregulation, respectively.

On the enrichment analysis, AD itself was identified as significant (Figure 3A). In the AD pathway, components related to the mitochondrial respiratory chain were downregulated, while components related to Ca^2+^ channels that transport Ca^2+^ from the ER into the cytoplasm were upregulated (Figure 3B). Several AD-related pathways, including proteasome, oxidative phosphorylation, and retrograde endocannabinoid signaling pathways, were also identified as statistically significant; most components in these pathways were downregulated (Figures 3C–E). Downregulation of the proteasome decreases the clearance of Aβ (Hong et al., 2014), while downregulation of oxidative phosphorylation decreases the efficacy of the mitochondrial respiratory chain, suggesting hypometabolism in the AD brain (Mosconi et al., 2008). The genes (e.g., *MFN1*, *OPA1*, and *DNM1L*) involved in mitochondrial fusion and fission were downregulated (Westermann, 2012), disturbing mitochondrial biogenesis to adopt energetic demands. The aberrant mitochondria were removed by the process, mitophagy. However, the genes responsible for mitophagy, *PINK1* and *PRKN* (Narendra et al., 2008), were downregulated, which further increased the burden of damaged protein clearance. The regulation of genes involved in oxidative phosphorylation in mitochondria was severely impaired in the present study, including genes for NADH dehydrogenase, succinate dehydrogenase, cytochrome *c* reductase, and cytochrome *c* oxidase in the electron transport chain. eCBs are signaling molecules used among nearby cells over short distances, and they modulate neuronal transmission. There is a high density of cannabinoid receptors in presynaptic terminals in the hippocampus and limbic cortex, indicating a role for cannabinoids in cognitive information processing (Zou and Kumar, 2018). A disruption of eCB signaling has been reported in AD, which influences neurotransmitter release (Basavarajappa et al., 2017). In the current study, most members of the retrograde endocannabinoid signaling pathway were downregulated, interfering with the role of eCBs in the release of glutamate and GABA in synapses.

### Subgroup Analysis

Among 77 comparisons, 15 investigated the amygdala and hippocampus of AD subjects, while 28 focused on the cerebral cortex (Table 5). Statistically significant DEGs for each subgroup analysis are shown in Supplementary File S4. There were 2,260 DEGs that were reported as significant in the amygdala and hippocampus, while 6,636 DEGs were reported as significant in the cerebral cortex. Among the 1,390 commonly found DEGs, 359 upregulated and 1,020 downregulated DEGs were consistently found in two subgroups. Eleven DEGs were upregulated in one subgroup but downregulated in another subgroup. The most highly upregulated and downregulated DEGs were *S100A6* and *STMN4*, respectively, in both subgroups. The protein calcyclin (encoded by *S100A6*) is a calcium-binding protein that is highly expressed in astrocytes surrounding Aβ deposits, and is involved in neuronal death via zinc depletion (Bartkowska et al., 2017). The protein stathmin (encoded by *STMN4*) binds tubulin to mediate microtubule dynamics, and dysregulation of stathmin-mediated microtubule stability induces memory loss (Uchida et al., 2014).

**TABLE 5:** The datasets used in the subgroup analysis.

Subgroups	No of control	No of disease	No of DEGs	No of pathways	Comparison_ID
Cerebellum	98	122	–	–	GSE44768
Putamen and nucleus accumbens	30	51	–	–	GSE84422_96_Putamen, GSE84422_97_Putamen, GSE84422_Nucleus_accumbens
Amygdala and hippocampus	175	159	2,260	18	GSE84422_Amygdala, GSE1297_CvsIni, GSE1297_CvsMod, GSE1297_CvsSev, GSE28146_CvsIni, GSE28146_CvsMod, GSE28146_CvsSev, GSE29378_CA1, GSE29378_CA3, GSE36980_HIP, GSE48350_HIP, GSE5281_HIP, GSE84422_96_Hippocampus, GSE84422_97_Hippocampus, GSE67333
Cerebral neocortices	632	849	6,636	25	E-MEXP-2280, GSE12685, GSE16759, GSE26927, GSE32645, GSE33000, GSE36980_FC, GSE36980_TC, GSE37263, GSE44770, GSE44771, GSE48350_EC, GSE5281_EC, GSE5281_PVC, GSE84422_96_Dorsolateral_prefrontal_cortex, GSE84422_96_Frontal_pole, GSE84422_96_Occipital_visual_cortex, GSE84422_96_Prefrontal_cortex, GSE84422_96_Superior_parietal_lobule, GSE84422_96_Temporal_pole, GSE84422_97_Dorsolateral_prefrontal_cortex, GSE84422_97_Frontal_pole, GSE84422_97_Occipital_visual_cortex, GSE84422_97_Prefrontal_cortex, GSE84422_97_Superior_parietal_lobule, GSE84422_97_Temporal_pole, GSE104704_Old, GSE53697

**FIGURE 4:**
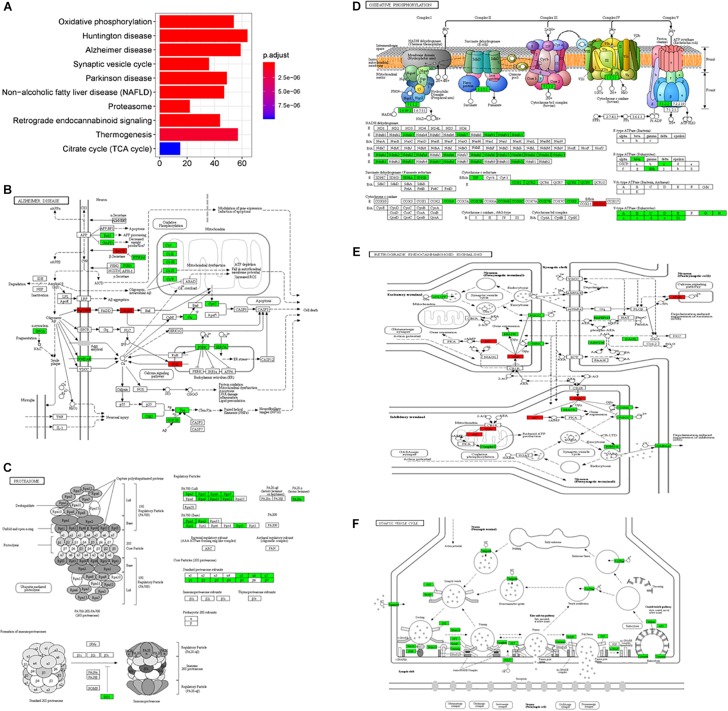
The AD-related biological pathways from the subgroup amydala and hippocampus, **(A)** the top 10 statistically significant pathways from enrichment analysis result; **(B)** Alzheimer’s disease; **(C)** Proteasome; **(D)** Oxidative phosphorylation; **(E)** Retrograde endocannabinoid signaling; and **(F)** Synaptic vesicle cycle. The red and green represent upregulation and downregulation, respectively.

**FIGURE 5:**
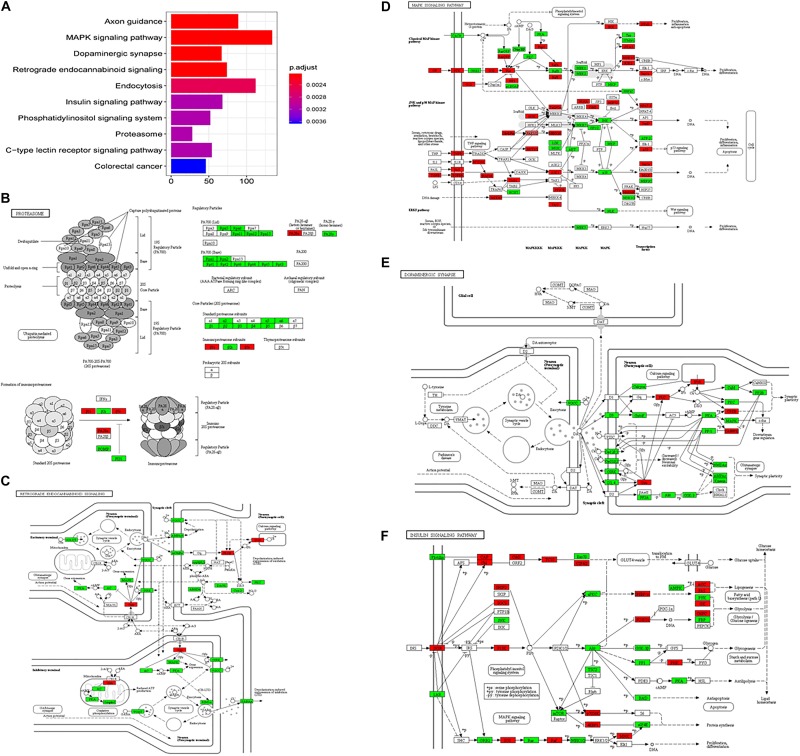
The AD-related biological pathways from the subgroup cerebral cortex, **(A)** the top 10 statistically significant pathways from enrichment analysis result; **(B)** Proteasome; **(C)** Retrograde endocannabinoid signaling; **(D)** MAPK signaling pathway; **(E)** Dopaminergic synapse; and **(F)** Insulin signaling pathways. The red and green represent upregulation and downregulation, respectively.

In the subgroup enrichment analyses, the pathways for proteasome and retrograde endocannabinoid signaling were found in both subgroups (Figures 4, 5). For the subgroup of the amygdala and hippocampus, the synaptic vesicle cycle was identified as statistically significant, and most components in this pathway were downregulated (Figure 4F). Downregulated synaptic functions decrease the release of neurotransmitters from neurons, inducing memory-related symptoms in AD. For the subgroup of the cerebral cortex, several synaptic signaling pathways were identified in AD, including the MAPK signaling pathway, dopaminergic synapses, and the insulin signaling pathway; the components in these pathways were mostly downregulated (Figures 5D–F). The role of the MAPK effector, JNK, is to dissociate Beclin 1 from Bcl-2 to mediate autophagy, and downregulation of JNK reduces autophagy ability (Zhou et al., 2015). The loss of dopaminergic synapses in the ventral tegmental area reduces the release of dopamine toward the cortex and hippocampus, and is reported to be associated with memory loss in AD before the formation of amyloid plaques (Nobili et al., 2017). Although all AD datasets collected in this study did not report suffering from T2DM, the dysfunctional insulin signaling was identified, suggesting an effect of Aβ in insulin signaling pathway. Aβ shares a common sequence with insulin, antagonizing the regulatory effects of insulin (Folch et al., 2018). In the canonical insulin signaling pathway, insulin binds receptors to activate Akt for mediating downstream effects, e.g., translocating glucose receptors to cellular membrane, activating mTOR for autophagy regulation, and inhibiting GSK3β for tau protein phosphorylation. Losing the regulatory effects of insulin may induce dysfunctional autophagy and tau protein hyperphosphorylation, potentiating AD development. The increased level of free insulin due to Aβ blockage reduces Aβ clearance via IDE, because insulin has a higher affinity to IDE than Aβ (Farris et al., 2003). On the other hand, the AD progression reduces the transport of insulin across BBB, decreasing insulin signaling in brain (Stanley et al., 2016).

In the present study, several datasets focused on the hippocampus and entorhinal cortex because these two regions suffer the most significant neuronal loss in the early stage of AD, while no significant neuronal loss is observed in these regions during normal aging. The transcriptome from these brain regions therefore represents the chronic response to the causes and consequences, or the treatment, of AD.

### Quality Assessment of Studies

**FIGURE 6:**
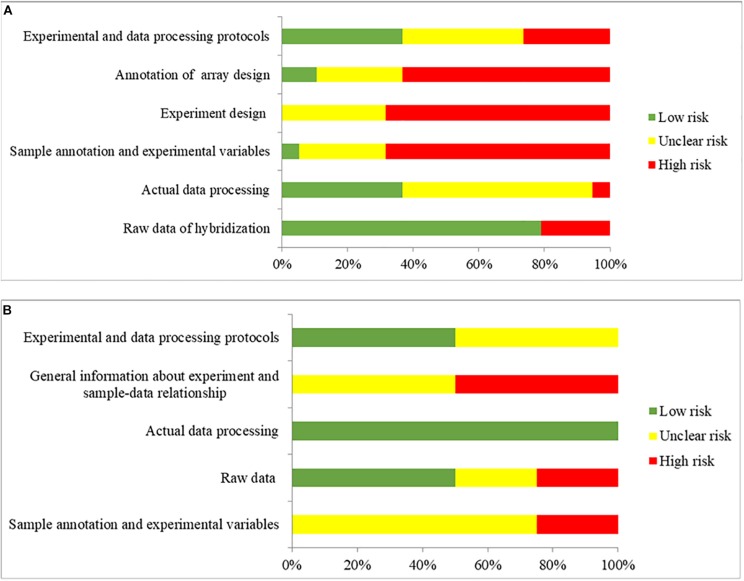
The overall quality assessment for **(A)** 19 studies for DNA microarray and **(B)** 4 studies for RNA-seq. Green color represents low risk of bias, which the authors clearly provided the information with full detail. Yellow color represents unclear risk of bias, which the authors provided the information without full detail. Red color represents high risk of bias, which the authors did not provide the correct information.

MIAME and MINSEQE guidelines were used to assess the transcriptomic analysis of studies (Blalock et al., 2004, 2011; Liang et al., 2007; Berchtold et al., 2008; Bronner et al., 2009; Williams et al., 2009; Nunez-Iglesias et al., 2010; Tan et al., 2010; Durrenberger et al., 2012; Antonell et al., 2013; Fischer et al., 2013; Janssen et al., 2013; Miller et al., 2013; Zhang et al., 2013; Hokama et al., 2014; Narayanan et al., 2014; Magistri et al., 2015; Scheckel et al., 2016; Wang et al., 2016; Lachen-Montes et al., 2017; Friedman et al., 2018; Nativio et al., 2018; Stopa et al., 2018) that published the datasets that were used in the present study. The results of quality assessments are shown in Figure 6. Among the 19 studies that published the microarray datasets, 80% did not provide the brain sample size or the weight used for RNA extraction for sample annotation and experimental variables, and only two studies provided sufficient information about experimental design, including quality control of samples. Furthermore, the annotations of array designs, including microarray quality indicators, were not fully reported for most studies (95%). For 40% of the studies, experimental and data processing details, including normalization methods and cut-offs for DEGs, were not fully reported. Among the four studies that published the RNA-Seq datasets, no study provided the brain sample size or the weight used for RNA extraction, or the details of experimental parameters used for RNA-Seq procedures. Furthermore, half of the studies did not provide sufficient information about experimental and data processing protocols, including the cut-offs for DEGs.

The relatively low quality of the studies indicates the insufficient information related to transcriptomic analysis provided in the studies, raising the reproducibility issue. Also, the transcriptomic results might be influenced, blurring some pathological events in the postmortem AD brains. The genes encoded heat shock proteins (e.g., *ATXN1*, *ATXN3*, and *ATXN7*) were not found statistically significant in the meta-analysis, while the multiple genes involved in neuroinflammation (e.g., *CXCL3*, *IFNG*, *IL6*, *IL13*, and *CXCL9*) (Garwood et al., 2011) were only reported as DEGs in one dataset, and therefore were not collected for meta-analysis. Although both heat shock proteins recruitment and neuroinflammation are associated with AD development, their pathological roles in AD development might not fully reflected in this study.

### Construction of a Protein–Protein Interaction Network to Identify Potential Pathogenic Factors

**FIGURE 7:**
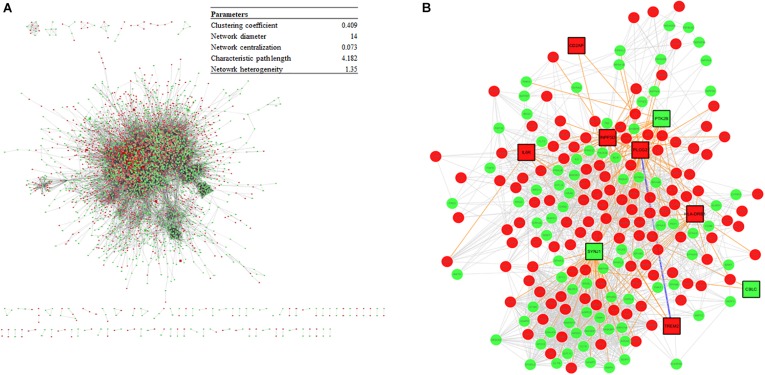
**(A)** The PPIN constructed from DEGs and the PPIN topology parameters. The red and green represent upregulation and downregulation, respectively. **(B)** A disease module, derived from the DIAMOnD algorithm, consisting of AD seed proteins (represented as squares) and their corresponding DIAMOnD proteins (represented as circles). The colors of the nodes represent upregulation (red), and downregulation (green). The edges represent seed-seed interaction (dark green), seed-DIAMOnD interaction (purple), and DIAMOnD-DIAMOnD interaction (pink).

The DEGs from the meta-analysis were used to retrieve the corresponding proteins in interactions according to STRING. The PPIN consisted of 4,781 nodes (2,083 upregulated nodes and 2,698 downregulated nodes) and 51,076 edges (Figure 7A) and there were 105 AD seed genes (Supplementary File S5) retrieved from the GWAS Catalog. The PPIN exhibited scale free topology with the degree distribution following a power law distribution (*y* = 3779.2x^–1^.^481^) and the most proteins were closely linked. In the PPIN, polyubiquitin-C (endcoded by *UBC*) and E3 ubiquitin-protein ligase RBX1 (encoded by *RBX1*), were the two proteins with the highest degree (372 and 222, respectively). These proteins were downregulated and are parts of UPS for protein degradation. The dysfunctional proteasome is regarded as an early event of AD (Manavalan et al., 2013), e.g., the decreased proteasomal clearance enhances mitochondrial dysfunction (Braun et al., 2015).

The PPIN for each protein pair was extracted for the subsequent DIAMOnD algorithm with AD seed genes to obtain a disease module. Not every seed gene was biologically linked to AD; some were involved in AD comorbidity disease. Among the disease modules (Figure 7B), the two nodes with a high degree (>95th percentile) were selected as potential pathogenic factors, and included EGFR and Fyn. The EGFR was the Aβ oligomer receptor, in which Aβ oligomer is regarded as the causative toxic species. The hydrophobic residues of Aβ oligomers are more easily accessible and interacting with cellular proteins, compared with Aβ fibrils (William et al., 2011). Fyn is involved in Aβ signal transduction and tau protein phosphorylation, and is changed in synaptic signaling pathways, including MAPK signaling pathway. These two proteins are both Aβ-relevant receptors, mediating the Aβ oligomers downstream neurotoxicity.

## Discussion

We used a meta-analysis approach based on DNA microarray and RNA-Seq datasets from multiple brain regions to reveal potential pathogenic factors of AD. Perturbations in the genes involved in the proteasome, oxidative phosphorylation, and retrograde endocannabinoid signaling pathways were identified. The proteasome is required to degrade the damaged proteins to maintain the cellular functionality. The decreased proteasome activity results in an accumulation of ubiquitinated and damaged proteins (Ciechanover, 2015). The ubiquitination degradation of APP by the proteasome includes several key genes, such as *PRKN*, *NEDD8*, *PSMC1*, *UCHL1*, and *UCHL3* (Bedford et al., 2008; Chen et al., 2012; Zhang et al., 2015; Nomura et al., 2016). Tau-specific ubiquitin–proteasome related genes, including *UBE2D2* (Shimura et al., 2004) and *STUB1* (Saidi et al., 2015), were also downregulated. The downregulation of these genes might result in impaired Aβ and phosphorylated tau protein clearance (Table 4). The oligomeric Aβ itself disrupts the catalytic activity of proteasome (Tseng et al., 2008), forming a positive feedback loop to worsen the Aβ clearance by proteasome. The disrupted catalytic activity of proteasome may also lose its inhibition of presenilin complex, which is the catalytic complex for γ-secretase, enhancing Aβ production (Chadwick et al., 2012). The accumulated Aβ, located on the mitochondria, deleteriously impairs oxidative phosphorylation, decreasing ATP production in the mitochondria (Chen and Du Yan, 2007; Moreira et al., 2010). The ATP-dependent proteins (i.e., the Ca^2+^ pumps located on the endoplasmic reticulum (ER) and ATP-dependent ion pumps) are unable to maintain their physiological functions, interfering neurotransmitter release and memory formation (Sudhof, 2012). The ATP-dependent molecular chaperones are also failed to work coordinately with UPS to remodel the misfolded proteins (Mattoo and Goloubinoff, 2014).

The reduced activity of proteasome enhances the burden of Aβ clearance on the autophagy and UPR. Autophagy is the process of degrading redundant cellular components by delivering them to lysosomes and forming autophagosomes. Beclin 1 (encoded by *BECN1*) is an essential component of autophagososomal structure (Jaeger and Wyss-Coray, 2010), but its autophagy function is inhibited by interacting with Bcl-2 (encoded by *BCL2*) leading to cellular death (Marquez and Xu, 2012). The downregulation of *BECN1* and upregulation of *BCL2* in the present study indicated autophagic flux failure. Autophagy is inhibited by the upregulation of *mTOR* and activated by the downregulation of *TFEB* (Caccamo et al., 2010; Polito et al., 2014). The downregulation of *mTOR* and upregulation of *TFEB* in the present study induced the formation of immature autophagosomes, impairing Aβ clearance. In addition, the downregulation of *mTOR* is associated with AD development (François et al., 2014), memory impairment (Slipczuk et al., 2009), and neuronal apoptosis (Yin et al., 2011). The depletion of autophagy-related genes, including *Atg5* and *Atg17*, impairs the elongation of phagopores to form autophagosomes, resulting in Aβ accumulation (Hara et al., 2006; Nilsson et al., 2013). Accumulated Aβ on the ER membrane induces ER stress, which is relieved by the activation of UPR. The activation of UPR is induced by the accumulation of misfolded proteins (Stutzbach et al., 2013), and has been reported in AD subjects (Scheper and Hoozemans, 2015). Two main UPR proteins, including PERK (encoded by *EIF2AK3*) and IRE-1 (encoded by *ERN1*), were upregulated and activated in AD due to misfolded Aβ oligomers (Joshi et al., 2016), although a recent critical review suggested that the involvement of ER stress in AD might be exaggerated by the misuse of APP/PS1-overexpressing AD mouse models (Hashimoto and Saido, 2018). The activation of UPR is a significant approach to prevent the accumulation of misfolded proteins, but may also increase Aβ production (Scheper and Hoozemans, 2015), and result in synaptic loss (Gong et al., 2016). Taken together, disturbed Aβ clearance could be the upstream event for AD pathological development, to induce Aβ accumulation, altering intracellular ionic gradients and resulting in insufficient oxidative phosphorylation.

The two top pathogenic factors determined in the present study, Fyn and EGFR, are key receptors in Aβ downstream signaling. Fyn is a protein tyrosine kinase belonging to the Src family, and it mediates synaptic plasticity in the central nervous system (Kaufman et al., 2015). The soluble Aβ oligomer binds to PrP^*C*^ with high affinity (Laurén et al., 2009), and interrupts the PrP^*C*^’s autoinhibitory mechanism (Wu et al., 2017), overactivating Fyn to increase glutamate release (Trepanier et al., 2012). Overactivation of Fyn, however, alters NMDA receptor function and intracellular Ca^2+^ homeostasis, rendering neurons vulnerable to Aβ-induced neurotoxicity. The Aβ-activated Fyn can phosphorylate downstream tau proteins at Tyr18 (Lee, 2004), and tau proteins participate in transporting Fyn to post-synaptic densities around glutamate receptors (Ittner et al., 2010). Fyn is a key protein that links between extracellular Aβ and intracellular tau protein, and unites these two pathologies in AD. The activity of Fyn is gene-dose-dependent (Um et al., 2012); that is, downregulation of *FYN* reduces Aβ-induced neurotoxicity, while upregulation of *FYN* exacerbates Aβ-induced neurotoxicity. In previous studies, overexpression of *FYN* has also been reported to accelerate synaptic loss, making Fyn inhibition a potential therapeutic treatment for AD (Chin et al., 2004; Chin, 2005). Saracatinib (AZD0530) is a Src kinase inhibitor that is being tested clinically for the treatment of AD. Saracatinib blocks Fyn activation to rescue memory deficits, and exhibits well-tolerated effects in mild-to-moderate AD subjects (Nygaard et al., 2015). A recently published clinical trial (VanDyck et al., 2019) revealed that saracatinib does not slow the decline in the CMRgl, and does not exhibit beneficial effects on several cognitive assessments, compared with the placebo group. However, a trend for slowing the reduction of volumetric measure in brain is observed, and a *post hoc* exploratory analysis deduces a statistically significant decline in CMRgl in the entorhinal cortex. Although the frequent adverse event of saracatinib, e.g., diarrhea, may limit the feasibility of increasing 125 mg daily to a higher dosage, the authors proposed that a higher dosage of saracatinib may be beneficial to slow decline of CMRgl in AD participants, who have greater tolerability.

**FIGURE 8:**
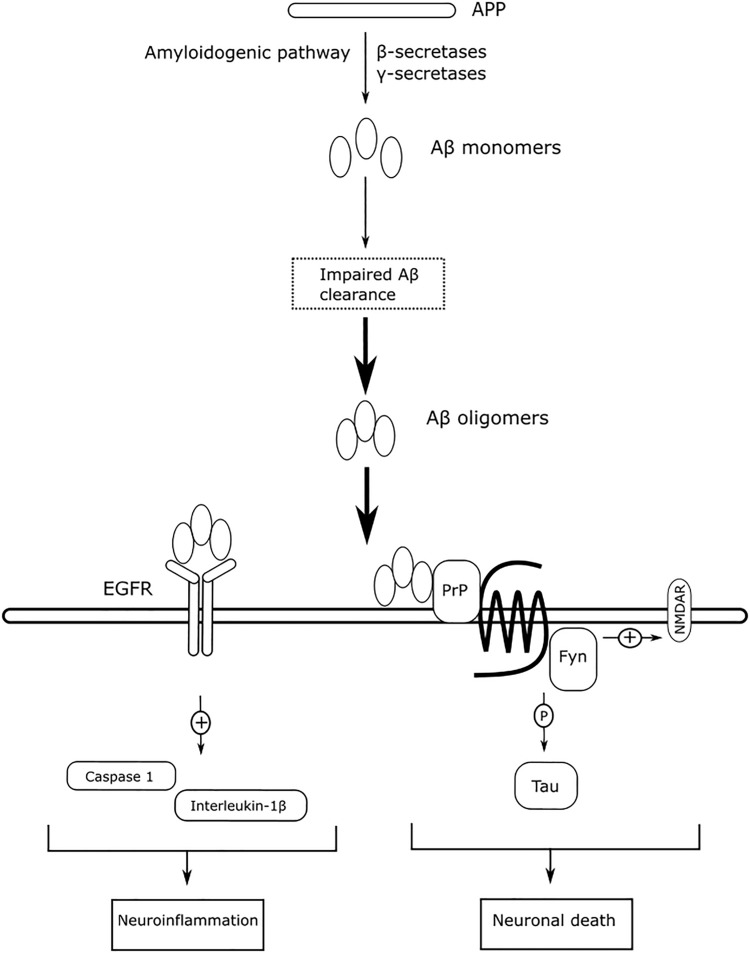
The mechanism of action of Aβ-mediated Fyn and EGFR in AD.

EGFR is more commonly associated with cancer than with AD. However, the expression of EGFR is statistically correlated with the expression of γ-secretase, suggesting a significant role of EGFR in AD (Zhang et al., 2007). EGFR is widely distributed in the hippocampus and cerebral cortex (Fu et al., 2003), and EGFR signaling exerts neurotrophic functions to regulate synaptic architecture in the central nervous system (Oyagi et al., 2011). The expression of *EGFR* is reported to be upregulated in the brains of AD animals, and overactivation of EGFR by Aβ oligomers induces memory loss (Wang et al., 2012). The overactivation of EGFR can also be induced by the prolong UPR (Papa and Germain, 2011). The levels of overactivated EGFR can be suppressed by treatment with the EGFR inhibitor gefitnib to rescue memory loss. Activation of EGFR also induces downstream neuroinflammatory cascades in response to Aβ-induced neurotoxicity. Afatinib, an orally available EGFR tyrosine kinase inhibitor, inhibits EGFR activation to alleviate neuroinflammation by reducing caspase 1 activation and interleukin-1β levels in neurodegenerative diseases (Chen et al., 2019). Antagonizing Aβ-induced activation of EGFR may have beneficial effects to slow memory loss and alleviate neuroinflammation. Figure 8 summarizes the mechanism of action of Aβ-mediated Fyn and EGFR in AD, as described above.

Besides the genes involved in Aβ downstream neurotoxicity, the genes related to amyloid clearance proteins (e.g., *ECE1* and *NEU1*) are reported as pathogenic factors of AD by other studies. Endothelin 1 (encoded by *ECE1*) is implicated in Aβ degradation and downregulation of *ECE1* enhances Aβ deposition (Pacheco-Quinto and Eckman, 2013). However, upregulation of *ECE1* was found in the present study, suggesting that the pathophysiological role of *ECE1* was not solely involved in Aβ metabolism, but also associated with the increase Aβ-induced vasoconstriction in AD (Palmer et al., 2013). Sialidase (encoded by *NEU1*) is a lysosomal enzyme for Aβ clearance through lysosomal exocytosis (Annunziata et al., 2013). Deficit of *NEU1* causes Aβ-induced proteasome inhibition, which was consistent with our results. Disruption of Aβ clearance proteins may be the secondary events of AD progression, rather than the primary cause. Increasing evidences show a correlation between T2DM and higher risk of developing AD, through increasing BBB permeability by chronic peripheral inflammation (Holmes et al., 2009). The BBB leakage was supported by the dysregulated genes in this study, including *OCLN*, *TJP1*, *CDH5*, and *CTNNB1*, which encode the tight junction proteins. The compromised BBB can be exacerbated by chronic hypertension (Santos et al., 2017) and microbial pathogens (Li H. et al., 2018), and alters peripheral immune cell trafficking to the brain and induces neuroinflammation. The neuroinflammation is mediated by p38 (encoded by *MAPK14*), which is a protein found in MAPK pathway. p38 is also a kinase to phosphorylate tau protein, making p38 as a potential pathogenic factor of AD. However, upregulation of p38 was not observed in this study, since p38 participates in numerous cellular events not only neuroinflammation. This can be supported by that although the inhibition of p38 alleviates neuroinflammation in AD (Yasuda et al., 2012; Ashabi et al., 2013), most p38 inhibitors are failed in clinical trials due to off-target effects. A gene converting glucose into lactate in glycolysis, *LDHA*, is regarded as a relevant pathogenic factor in AD, although it may not be specific to AD pathogenesis. Overexpression of *LDHA* is reported to prevent neurons from Aβ neurotoxicity by shifting mitochondrial glucose metabolism to lactate production, and decreasing ROS production (Newington et al., 2012). However, elevated lactate level may be detrimental to neurons (Harris et al., 2016). Although the expression of *LDHA* was insignificantly downregulated in this study, the opposing neuroprotective and neurotoxic effects of *LDHA* suggested that neurons might have its own defense mechanism against Aβ in the early onset of AD.

The strength of this study is the integration of transcriptomic data from both DNA microarrays and RNA-Seq using a systematic search and consistent workflow. However, there are some limitations. First, the articles that published the datasets did not provide full details of the experimental procedures, making the transcriptomic results less reliable and more difficult to repeat. In addition, the transcriptomic data retrieved from postmortem brain tissue using different methods might be biased. For example, laser-microdissection mainly targets neuronal soma, but may miss transcripts transporting to the pre- or post-synapses. Lacking of sufficient information about disposal of transcriptomic analysis, resulting in relatively low quality of the selected studies, might raise a reproducibility issue. Second, the confounding factors of postmortem brain tissues include age, sex, and postmortem interval, and may blur the results, e.g., neuroinflammation. Third, most included datasets in this study focused on hippocampus and entorhinal cortex, in which the brain region AD are believed to start. However, AD might start differently from those two brain regions, e.g., the impaired UPS as an early AD event was reported in brainstem (Irmler et al., 2012). Transcriptomic analysis in different regions of postmortem AD brains would provide more novel insights of AD development.

## Conclusion

This meta-analytical study suggested that the reduced Aβ clearance in AD pathogenesis was associated with the genes encoding Fyn and EGFR, which were key receptors in Aβ downstream signaling.

## Data Availability Statement

The raw data of this paper are available in the public database, GEO and ArrayExpress. The data that support the findings of this paper are available in the published article and its additional files.

## Author Contributions

SL and SY conceived and designed the study and wrote the manuscript. SY and HZ extracted and analyzed the data. SL and HZ verified the data and revised the manuscript. All authors read and approved the final version of the manuscript.

## Conflict of Interest

The authors declare that the research was conducted in the absence of any commercial or financial relationships that could be construed as a potential conflict of interest.

## Footnotes

1 https://www.ncbi.nlm.nih.gov/geo

2 https://www.ebi.ac.uk/arrayexpress/

3 http://www.genome.jp/kegg/

4 https://string-db.org/

5 https://www.ebi.ac.uk/gwas/

6 http://fged.org/projects/minseqe/

## Supplementary Material

FILE S1 The search strategy used in the database GEO and ArrayExpress.

FILE S2 The DEGs from each comparison.

FILE S3 The DEGs after meta-analysis found statistically significant in this study.

FILE S4 The statistical significant DEGs found in each subgroup.

FILE S5 The AD seed genes used in the DIAMOnD algorithm.

## Abbreviations

Aβ: amyloid- β
AD: Alzheimer’s disease
BBB: blood-brain barrier
CMRgl: cerebral metabolic rate for glucose
DEGs: differentially expressed genes
DIAMoND: DIseAse Module Detection algorithm
eCBs: endocannabinoids
FDR: false discovery rate
IDE: insulin-degrading enzyme
KEGG: Kyoto Encyclopedia of Genes and Genomes
logORs: log_e_ odds ratios
PPIN: protein–protein interaction network
PrP^*C*^: cellular prion protein
ROS: reactive oxygen species
RNA-Seq: RNA-sequencing
T2DM: type 2 diabetes mellitus
UPR: unfolded-protein response
UPS: ubiquitin-proteasome system.
